# Supplementary material for: Spatial evolution, influencing factors and spillover effects of logistics resilience in the Yangtze River Economic Belt
Source: PLoS One. 2024 Aug 22;19(8):e0303639. doi: 10.1371/journal.pone.0303639 (PMC11341063; doi:10.1371/journal.pone.0303639)
Supplement: Attachment — Submitted filename: Responses to reviewers_PONE-D-23-16786.docx [file pone.0303639.s001.docx]

**Responses to** **reviewer/editorial comments on the manuscript entitled**

***“Spatial evolution, influencing factors and spillover effects of logistics resilience in the Yangtze River Economic Belt”***

**Re-submitted to *PLOS ONE***

**Manuscript ID PONE-D-23-16786**

Dear editors and reviewers:

Thank you for your letter and for the reviewer/editorial comments concerning our manuscript entitled “*Spatial evolution, influencing factors and spillover effects of logistics resilience in the Yangtze River Economic Belt” (ID:PONE-D-23-16768).*

Those comments are all valuable and very helpful for revising and improving our paper, as well as the important guiding significance to our research. We have studied comments carefully and have made correction which we hope will meet with your approval. All revised portions are marked in red, and the deleted parts have been marked in the track changes mode in the revised manuscript which we would like to submit for your kind consideration.

The main corrections are in the manuscript and the responds to the editorial and reviewer’ s comments are as follows:

**Response to the editorial comments:**

1. *Please ensure that your manuscript meets PLOS ONE' s style requirements, including those for file naming. The PLOS ONE style templates can be found at*

*[https://journals.plos.org/plosone/s/file?id=wjVg/PLOSOne_formatting_sample_main_body.pdf](https://journals.plos.org/plosone/s/file?id=wjVg/PLOSOne_formatting_sample_main_body.pdf) and*

*[https://journals.plos.org/plosone/s/file?id=ba62/PLOSOne_formatting_sample_title_authors_affiliations.pdf](https://journals.plos.org/plosone/s/file?id=ba62/PLOSOne_formatting_sample_title_authors_affiliations.pdf)*

**Response:**

Thank you for pointing out our mistakes. We have made format adjustment for the revised manuscript according to PLOS ONE' s style requirements. Please see the revised manuscript.

2. *Thank you for stating the following financial disclosure: "Yin Huang:this study was supported by the National Natural Science Foundation of China (Grant no.72174214); Key Project of Scientific Research of Department of Education of Hunan Province (Grant no. 21A0156). Xiaofan Zhang: this study was supported by Hunan Provincial Innovation Foundation for Postgraduate (Grant no. CX20220737)." Please state what role the funders took in the study.  If the funders had no role, please state: "The funders had no role in study design, data collection and analysis, decision to publish, or preparation of the manuscript." If this statement is not correct you must amend it as needed. Please include this amended Role of Funder statement in your cover letter; we will change the online submission form on your behalf.*

**Response:**

Thanks for your suggestion. We have made a statement about the funder role in our cover letter and explained the roles of authors on the title page of the manuscript. Please see the cover letter and the title page.

3. *In your Data Availability statement, you have not specified where the minimal data set underlying the results described in your manuscript can be found. PLOS defines a study's minimal data set as the underlying data used to reach the conclusions drawn in the manuscript and any additional data required to replicate the reported study findings in their entirety. All PLOS journals require that the minimal data set be made fully available. For more information about our data policy, please see [http://journals.plos.org/plosone/s/data-availability](http://journals.plos.org/plosone/s/data-availability).*

**Response:**

Thank you for pointing out our mistakes. We have uploaded the minimal data set for the original calculation as the supporting file. Please see the supporting files.

4. *We note that Figures 1, 2, 3, and 4 in your submission contain [map/satellite] images which may be copyrighted. All PLOS content is published under the Creative Commons Attribution License (CC BY 4.0), which means that the manuscript, images, and Supporting Information files will be freely available online, and any third party is permitted to access, download, copy, distribute, and use these materials in any way, even commercially, with proper attribution. For these reasons, we cannot publish previously copyrighted maps or satellite images created using proprietary data, such as Google software (Google Maps, Street View, and Earth). For more information, see our copyright guidelines: [http://journals.plos.org/plosone/s/licenses-and-copyright](http://journals.plos.org/plosone/s/licenses-and-copyright)*

*Please include captions for your Supporting Information files at the end of your manuscript, and update any in-text citations to match accordingly. Please see our Supporting Information guidelines for more information: [http://journals.plos.org/plosone/s/supporting-information](http://journals.plos.org/plosone/s/supporting-information).*

**Response:**

We uploaded the written permission from the copyright holder of the maps used in Fig 1 and Fig 2, and inserted the source information of the maps used in these images in the form of reference in the caption of Figures 1 and 2.

5. *Please include captions for your Supporting Information files at the end of your manuscript, and update any in-text citations to match accordingly. Please see our Supporting Information guidelines for more information:*

*[http://journals.plos.org/plosone/s/supporting-information](http://journals.plos.org/plosone/s/supporting-information).*

**Response:**

Thank you for pointing out our mistakes. We have added supporting information at the end of the manuscript. Please see the revised manuscript.

6. *Please review your reference list to ensure that it is complete and correct. If you have cited papers that have been retracted, please include the rationale for doing so in the manuscript text, or remove these references and replace them with relevant current references. Any changes to the reference list should be mentioned in the rebuttal letter that accompanies your revised manuscript. If you need to cite a retracted article, indicate the article’s retracted status in the References list and also include a citation and full reference for the retraction notice.*

**Response:**

Thanks for your suggestion. We have edited the reference list and checked the citation format of the references to ensure that they meet the format requirements of *PLoS ONE.*

**Response to the comments from reviewer 1:**

*1. The innovation of this paper needs to be highlighted in the abstract. The abstract need to rewrite.*

**Response:**

Thanks for your suggestion. We have rewritten the abstract, mainly expounding the importance of studying logistics resilience and the innovation points of this paper in the research perspective, logistics resilience evaluation system design and data selection. Due to the word limit of the abstract, we introduce the innovation of the research perspective, research method and data selection in detail in the discussion part of the paper. First, based on the perspective of economic geography, a spatial econometric model is constructed to analyze the influencing factors that lead to the differences in logistics resilience of different cities and the existing spatial spillover effect. Second, in the design of logistics resilience rating system, considering the market, external environment and ecology, the urban logistics resilience is evaluated from the supply and demand relationship, industrial structure and the impact on the environment. Third, in terms of data selection, this paper comprehensively selects statistical data and POI data into the model for calculation, and makes an innovative attempt in data selection. **Please see the abstract in the revised manuscript.**

*2. The authors need to add the background of this research.*

**Response:**

Thank you for your proposal on adding the background of this research. First, we revised the introduction, emphasizing that the research background of this paper is the post-COVID-19 era, explaining the important role of the logistics industry and logistics resilience in economic recovery after the COVID-19 pandemic, and introducing the geographical location of the Yangtze River Economic Belt. Second, we have made major revisions to the literature review. In part 2.2 "Study on logistics industry and logistics resilience in the Yangtze River Economic Belt", the development status of regional development imbalance in the Yangtze River Economic Belt is introduced, the important role of regional logistics construction for high-quality and sustainable development of regional economy is expounded, and the shortcomings of previous studies on logistics resilience in the Yangtze River Economic Belt are summarized. **Please see the introduction and literature review in the revised manuscript.**

*3. The literature review is not enough, the innovation of this paper and the contribution made by previous studies have not been clearly expressed. The author should summarize the existing research gaps and highlight the innovation of this paper after completing the literature review.*

**Response:**

Thank you for pointing out our mistakes. We have made a major revision to the literature review, which is divided into three parts.

(1) In section 2.1 "Study on resilience and logistics resilience", we sorted out the definition of the word "resilience" and the process of concept expansion, summarized the relevant definitions of supply chain resilience and logistics resilience, and presented them in Table 1 of the revised manuscript. By reviewing and summarizing the research on supply chain resilience and logistics resilience, we define logistics resilience.
(2) We changed the second part of the literature review to "Study on logistics industry and logistics resilience in the Yangtze River Economic Belt", reviewed relevant research on the development of the logistics industry in the Yangtze River Economic Belt, and highlighted the important role of the logistics industry in the high-quality and sustainable development of the Yangtze River Economic Belt.
(3) We summarized the shortcomings of the existing studies on the logistics resilience of the Yangtze River Economic Belt, and introduce the main research content and innovation points of this paper. **Please see the literature review in the revised manuscript.**

*4. It is suggested that the authors add more descriptions of the reasons for choosing Yangtze River Economic Belt as a case study, the following literature should be helpful for your research：(1) Adaptability analysis of water pollution and advanced industrial structure in Jiangsu Province, China. (2)Coordination of the Industrial-Ecological Economy in the Yangtze River Economic Belt, China. (3)Compilation of Water Resource Balance Sheets under Unified Accounting of Water Quantity and Quality, a Case Study of Hubei Province.*

**Response:**

Thanks for your suggestion on adding more descriptions of the reasons for choosing Yangtze River Economic Belt as a case study. Referring to the several literature you listed, we have added descriptions of the development status of the Yangtze River Economic Belt in the introduction and literature review, which is also the reason why this paper chooses the Yangtze River Economic Belt as the study region. **Please see the references in the revised manuscript.**

5. *The author needs to explain the meaning of logistics resilience and explain the reasons why it includes four aspects: logistics supply, logistics demand, industrial structure, and impact on environment.*

**Response:**

We agree with the reviewer’s opinions on explaining the meaning of logistics resilience and the design idea of logistics resilience evaluation system. First, in the part of literature review, we defined the concept of logistics resilience by reviewing the existing studies on supply chain resilience and logistics resilience. Logistics resilience is defined as the resistance of the regional logistics system when it is disturbed by external economic, natural and other environmental conditions, or the recovery and improvement ability of the regional logistics system to a better path through system optimization. Second, in the method part, it explains the reasons and background of choosing these four angles to measure logistics resilience: (1) Logistics supply and logistics demand: logistics supply and demand have a profound impact on the performance of the logistics market, which further affects the resilience of logistics. (2) Industrial structure: as a service industry, logistics is closely related to other industries, especially manufacturing, so changes in industrial structure will also affect logistics resilience by affecting logistics demand. (3) Impact on environment: transportation activities in the logistics industry are the main source of environmental pollution, and their impact on the ecological environment will eventually affect the sustainable development of the logistics industry through the feedback mechanism. Therefore, paying attention to the impact of the development of the logistics industry on the ecological environment is of great significance for the evaluation and improvement of logistics resilience. **Please see the literature review and methodology in the revised manuscript.**
