# Supplementary material for: Spatial evolution, influencing factors and spillover effects of logistics resilience in the Yangtze River Economic Belt
Source: PLoS One. 2024 Aug 22;19(8):e0303639. doi: 10.1371/journal.pone.0303639 (PMC11341063; doi:10.1371/journal.pone.0303639)
Supplement: Attachment — Submitted filename: Responses to reviewers_PONE-D-23-16786R1.docx [file pone.0303639.s002.docx]

**Responses to** **reviewer/editorial comments on the manuscript entitled**

***“Spatial evolution, influencing factors and spillover effects of logistics resilience in the Yangtze River Economic Belt”***

**Re-submitted to *PLOS ONE***

**Manuscript ID PONE-D-23-16786R1**

Dear editors and reviewers:

Thank you for your letter and for the reviewer/editorial comments concerning our manuscript entitled “*Spatial evolution, influencing factors and spillover effects of logistics resilience in the Yangtze River Economic Belt” (ID:PONE-D-23-16768R1).*

Those comments are all valuable and very helpful for revising and improving our paper, as well as the important guiding significance to our research. We have studied comments carefully and have made correction which we hope will meet with your approval. All revised portions are marked in red in the revised manuscript which we would like to submit for your kind consideration.

The main corrections are in the manuscript and the responds to the editorial and reviewer’ s comments are as follows:

**Response to the comments from reviewer 1:**

*1. In the "Abstract" section, it is better to describe the research by mentioning the quantitative results of study. Readers should be able to see the quantities of results in the abstract. the abstract should also contain Objectives, Methods/Analysis, Findings, and Novelty /Improvement.*

**Response:**

Thanks for your suggestion. We have rewritten the abstract, mainly adding the quantitative results of this study and the possible directions for future improvement. Due to the word limit of the abstract, we introduce the innovation of the research perspective, research method and data selection in detail in the discussion part of the paper. First, based on the perspective of economic geography, a spatial econometric model is constructed to analyze the influencing factors that lead to the differences in logistics resilience of different cities and the existing spatial spillover effect. Second, in the design of logistics resilience rating system, considering the market, external environment and ecology, the urban logistics resilience is evaluated from the supply and demand relationship, industrial structure and the impact on the environment. Third, in terms of data selection, this paper comprehensively selects statistical data and POI data into the model for calculation, and makes an innovative attempt in data selection. **Please see the abstract in the revised manuscript.**

*2. In the "Introduction" section, the introduction expounds too much very general and well-known information, and the importance and significance of the research are not highlighted.*

**Response:**

Thank you for your valuable suggestion. The introduction explains the significance of this study from three perspectives: the importance of logistics industry to global economic recovery, the significance of logistics resilience in the process of regional sustainable development and the imbalance of logistics industry development in the Yangtze River Economic Belt. We have deleted part of the introduction to make it more concise and to better highlight the practical significance of conducting this research. **Please see the introduction in the revised manuscript.**

3. *In the "Introduction" section, The author lacks references when expounding the research background.*

**Response:**

Thank you for your proposal on adding more references in the “Introduction” section. We have removed parts of the introduction and made appropriate citations in existing references in the article. **Please see the introduction in the revised manuscript.**

4. *For readers to quickly catch your contribution, it would be better to highlight major difficulties and challenges, and your original achievements to overcome them, in a clearer way in abstract and introduction.*

**Response:**

Thanks for your suggestion. We have rewritten the abstract, mainly adding the quantitative results of this study and the possible directions for future improvement. At the same time, we also explain the data sources of this study in the abstract, corresponding to the innovation of this paper mentioned at the end of the paper. **Please see the abstract in the revised manuscript.**

5. *More explanation is needed for where there is a research gap and what the goals of the research are. The research gap and the goals of the research are not explained in detail which leads to the reader missing the significance of the research.*

**Response:**

We agree with the reviewer’s opinions on explaining the research gap and research goal of this study. In the literature review, by combing the relevant literature of resilience research, we summarized the gaps in current research and expounded the significance of carrying out this research. At the end of the literature review, we inserted a research framework diagram to explain the purpose and main contents of this study. **Please see the summary of the literature review and the research framework in the revised manuscript.**

6. *In the second section, Please provide a framework diagram of this paper.*

**Response:**

Thanks for your suggestion on adding a framework diagram of this paper. At the end of the literature review, we add the framework diagram of this paper. **Please see the research framework in the revised manuscript.**

7. *The analysis of the results is very superficial. It simply describes the change trend of the data, and lacks in-depth analysis and interpretation.*

**Response:**

Thanks for your suggestion on adding in-depth analysis and interpretation. First of all, In the part of logistics resilience measurement, we separately explain the calculation results of logistics resilience of several special cities by giving examples, and emphasize the change of logistics resilience of the Yangtze River Economic Belt over time to highlight the fine granularity of the study, so as to make our research results more typical. Second, the spatio-temporal distribution of logistics resilience is the foundation for subsequent research, rather than the main part of this study. We substitute the calculation results of logistics resilience into the subsequent spatial Durbin model as explained variables to calculate the influence and spillover effect of different factors on logistics resilience. Therefore, considering the word limit of the article, we did not give a very detailed describe the distribution characteristics of logistics resilience. Finally, in our original manuscript, we first described the calculated quantitative results in the part of impact factor analysis and spillover effect measurement, and then analyzed the possible reasons for the results. The relevant content of the result analysis is highlighted in red. **Please see the results in the revised manuscript.**

8. *How can your study inspire studies on other regions? The results should be further elaborated to show how they could be used for the real applications.*

**Response:**

Thanks for your suggestion on elaborating how the results could be used for the real applications. In the suggestion section, we discussed the aspects in which cities in the Yangtze River Economic Belt should strengthen construction to promote their own logistics resilience according to the influencing factors and spillover effect analysis results. As for the implications of this study for other regions, we believe that the Yangtze River Economic Belt connects developed and less developed regions, and it can be seen as a microcosm of a country, and explain how other regions can take the spatial spillover effect between cities in the Yangtze River Economic Belt to improve their own logistics resilience. **Please see the suggestions in the revised manuscript.**

9. *The article lacks an important discussion link, in which the author should focus on describing the differences between the article study and other scholars' studies, thus highlighting the relevance and academic value of the article, the following literature should be helpful for your research: (1)Reduction pathways identification of Agricultural Water Pollution in Hubei Province, China. (2) A differential game of water pollution management in the trans-jurisdictional river basin\.*

**Response:**

Thanks for your suggestion. In the discussion section at the end of the paper, we put forward the innovation in the research perspective, research methods and data sources by comparing the existing researches. In the conclusion part, we summarized the limitations of this study and the direction for improvement in the future by comparing the studies of other scholars. We cited a reference you provided, which provided us with a feasible research method for future research. **Please see discussion and conclusions in the revised manuscript.**

10. *The article was not written following the correct journal's guidelines to be considered for publication. INTORDCUTION→METHOD→RESULTS→DISSCUSION→CONCLUSION.*

**Response:**

Thank you for your reminding. As for the literature review, by combing the relevant literature of resilience research, we found the gap of current research, which is the basis of this study. We adopted the comments of the first review, and added a summary of the existing studies in the literature review. Based on this, we explained the objectives of this study and highlighted the purpose and significance of this study. Therefore, we did not delete the literature review. We have changed the title of the second half of the article according to your suggestion**.**
